# Second Version of Google Glass as a Wearable Socio-Affective Aid: Positive School Desirability, High Usability, and Theoretical Framework in a Sample of Children with Autism

**DOI:** 10.2196/humanfactors.8785

**Published:** 2018-01-04

**Authors:** Ned T Sahin, Neha U Keshav, Joseph P Salisbury, Arshya Vahabzadeh

**Affiliations:** ^1^ Brain Power Cambridge, MA United States; ^2^ Department of Psychology Harvard University Cambridge, MA United States; ^3^ Massachusetts General Hospital Boston, MA United States

**Keywords:** autism, technology, digital health, augmented reality, virtual reality, smartglasses, usability, schools, education, classroom, IDEA, IEP, special education

## Abstract

**Background:**

Computerized smartglasses are being developed as an assistive technology for daily activities in children and adults with autism spectrum disorder (ASD). While smartglasses may be able to help with educational and behavioral needs, their usability and acceptability in children with ASD is largely unknown. There have been reports of negative social perceptions surrounding smartglasses use in mainstream populations, a concern given that assistive technologies may already carry their own stigma. Children with ASD may also have a range of additional behavioral, developmental, and social challenges when asked to use this emerging technology in school and home settings.

**Objective:**

The usability and acceptability of Glass Enterprise Edition (Glass), the successor to Google Glass smartglasses, were explored in children with ASD and their caregivers.

**Methods:**

Eight children with ASD and their caregivers were recruited to attend a demonstration session with Glass smartglasses the week they were publicly released. The children had a wide range of ability, including limited speech to speaking, and represented a full range of school ages (6 to 17 years). Children and caregivers were interviewed about their experience of using the smartglasses and whether they would use them at school and home.

**Results:**

All 8 children succeeded in using Glass and did not feel stressed (8/8, 100%) or experience any overwhelming sensory or emotional issues during the session (8/8, 100%). All 8 children (8/8, 100%) endorsed that they would be willing to wear and use the device in both home and school settings. Caregivers felt the experience was fun for the children (8/8, 100%), and most caregivers felt the experience was better than they had expected (6/8, 75%).

**Conclusions:**

A wide age and ability range of children with ASD used Glass immediately after it was released and found it to be usable and acceptable. Despite concerns about potential stigma or social acceptability, all of the children were prepared to use the technology in both home and school settings. Encouragingly, most caregivers noted a very positive response. There were no behavioral, developmental, or social- or stigma-related concerns during or after the session. Smartglasses may be a useful future technology for children with ASD and are readily accepted for use by children with ASD and their caregivers.

## Introduction

### Background

Autism spectrum disorder (ASD) is a childhood-onset developmental disorder, with an estimated 3.5 million people being diagnosable with ASD in the United States alone [1]. Innovative assistive technologies may help to address the unmet educational and therapeutic resource demands of the ASD community [2]. While there are many different types of assistive technology, the portability, capability, and ubiquity of smartphone and tablet devices has led to considerable growth in assistive apps for these devices [3,4]. More recent technological advances have resulted in lightweight smartglasses: face-worn computers with a visual display and in-built sensors [5-7] that can also deliver assistive apps [8,9].

Smartglasses can deliver a large range of experiences, including augmented and virtual reality [10]. They are also sensor-rich and can collect a wide range of quantitative user data [9,11,12]. These data can be monitored and analyzed on a real-time basis, allowing for the smartglasses to dynamically change the user experience to optimize learning—effectively placing the user and the smartglasses in a closed feedback loop [8,13,14]. Given the proximity of smartglasses to the sensory organs contained in the human head, this type of computing may enable a higher level of human-computer interaction than other devices [13]. Smartglasses are already being developed as a social and behavioral communication aid for people with ASD [8,15,16].

**Figure 1:**
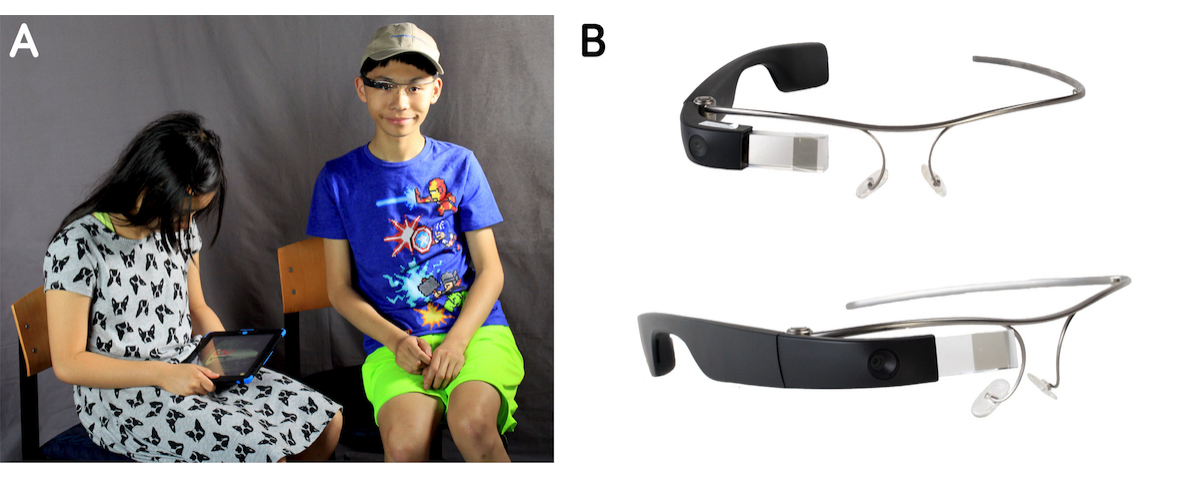
Head-worn computers encourage users to be heads-up and allow them to be hands-free in contrast to screen-based technologies such as phones and tablets. (A) Demonstrative example of a person using a tablet while her sibling uses Glass Enterprise Edition, days after it was released. Both siblings have autism spectrum disorder. Tablet use encourages a heads-down stance, suboptimal posture, and visual disconnection from the social world. (B) The Glass Enterprise Edition device from multiple views.

There are a number of important differentiating factors to consider when smartglasses are compared to handheld devices. Handheld devices such as tablets and smartphones require one or both hands to hold the device and encourage a heads-down posture (Figure 1 A, left) [17]. Evidence suggests that smartphone use may decrease user awareness of their social and physical environment. This is a particular concern in people with ASD, given that they already often face challenges engaging with the social world around them [18]. In contrast, head-worn computers pose an advantage in allowing and potentially encouraging children to remain heads-up while using them. This heads-up posture when using smartglasses can allow for better user engagement with people and the social world (Figure 1 A, right).

### Modern Assistive-Reality Smartglasses

The emergence of a new crop of smartglasses is encouraging, especially because the initial public reaction to the widely recognized original Google Glass resulted in some negative social reactions. Modern smartglasses vary in terms of physical dimensions, functionality, and intended user group. For the purposes of this report, we decided to investigate the acceptability and usability of the most recently released lightweight smartglasses, Glass Enterprise Edition (Glass). Glass was released by X (a subsidiary of Alphabet Inc, formerly known as Google X) in July 2017. Glass is an assistive-reality technology, and it is the successor to Google Glass, one of the most recognizable smartglasses in the world [19]. Glass, like its predecessor, is a head-mounted, wearable computer that has demonstrated utility in a variety of situations where operating a computer hands-free and while heads-up is of particular advantage. Glass has been creatively developed as a technology that can deliver social and cognitive skills coaching to children and adults with ASD [8]. To our knowledge, we have reported on the first studies of ASD-related software on the original Google Glass (Explorer Edition) [8,9,15,16], and here we present the first appearance of Glass (Enterprise Edition) in the literature.

It would seem that the Enterprise Edition (which has updates to the form factor, usability, central processor, display, audio system, and other features) would represent a substantial advantage for assistive technology apps and algorithms for ASD. However, it remains unknown whether people with ASD would actually desire to wear the new device. Assistive apps for people with ASD on the original Google Glass have been shown to be tolerable [20], safe [15], and to reduce hyperactivity in an ASD sample [8,16]. However, small changes in devices can greatly affect the desire of potential users to wear them.

Given that the initial entry of Google Glass and other smartglasses raised privacy concerns and some negative public reaction, the announcement of a major new release of head-worn computing [19] signaled a potentially major advance for assistive technology targeting populations who traditionally face significant social challenges [17]. Google Glass was ahead of its time and may have been held back by perceptions around desirability and social acceptability of wearing this new category of device in public [21,22]. It is therefore reassuring to developers that head-worn computer platforms have received public backing from one of the largest companies in the world [19], in this case the inventor of the product [23].

### Understanding the Needs of People With Autism Spectrum Disorder

As with any assistive technology, it is important to investigate and understand the attitudes of children and young adults with ASD, especially because children with special needs are often forced to use devices and systems they do not actually like or want to be associated with [24,25]. This is ultimately less effective because aversion leads to lower adherence. Poor adherence and problems with maintaining lasting engagement are some of the largest issues facing educational devices and apps as well as well-being and lifestyle tools [26,27].

Many people with ASD use assistive technology to help them with communication skills, social and emotional skills, and adaptive/daily activities and living skills [28]. Assistive technologies elicit a range of responses from individuals and their peers, and they can be considered cool [25], weird, desirable, or a source of stigma [29,30]. Users of assistive technologies can often express a preference for the type of assistive technology that they want to use [31,32], even at a young age [33]. Additionally, the social acceptability of an assistive technology may be one of the most important elements in determining if that technology gets used by people with developmental disabilities [30,34]. These individuals have often had to use technologies that have been selected for them and their families while having little input to the potential negative image, stigma, or embarrassment of using such technologies [30]. Understanding and implementing user preference of assistive technologies empowers self-determination in these individuals [31]. The preferences and views of the family and caregivers of these individuals are also important as they impact the acceptance and effective use of such technologies in the household [28,35]. These issues are pertinent to smartglasses in light of past reports of negative public perception (eg, around privacy concerns [22]).

There have only been a handful of reports on the use of smartglasses in people with ASD [8,15,16], and the attitudes toward and acceptability of such devices to people with ASD remains unclear. The use of smartglasses in people with ASD also requires discussion of their potential impact on social communication from a cognitive neuroscience standpoint and their prospective influence on child development from ecological, psychosocial, and cognitive child development theories.

### Potential Impact of Smartglasses on Social Communication

The human face, a complex and dynamic system, is our most powerful means of social communication [36]. To successfully transmit social information to another person, the sender must have the mental and physical means of generating a facial and bodily representation of the social information that she or he wishes to send, while the receiver must be in a position to see and decode the facial and bodily representations into social information. The social communication deficits seen in ASD may impede the ability to send and receive social information. People with ASD are reported to have deficits in facial perception [37,38], emotion recognition [39], eye gaze [40], and production of facial expressions [41]. It is important to consider the possibility that social communication may be further impacted by the physical presence of smartglasses on a sender’s face. Smartglasses may impede social communication if, for example, the sender demonstrates a hesitancy in producing natural head movements or expressing large magnitude facial emotional expressions due to concern that the smartglasses may fall off the face or be damaged. Smartglasses may also impair social interaction if the user feels the assistive device is socially undesirable [42] or a source of stigma [30]. In these situations, users may not use the device or may alter their facial and bodily actions to minimize attention to themselves. Furthermore, the physical form factor of smartglasses may obscure a portion of the wearer’s face that is visible to others, especially the central information-rich parts of the face such as the eye regions [43].

The relative effect of this obscuring of the facial region may be dependent on the size of the individual’s face relative to the smartglasses, which may correlate with the age of the individual given that biologic age determines an individual’s head size [44]. It may also depend on the ability of the receiver to successfully compensate for partly missing facial data and to make inferences about a sender (a common application of this in ASD research is the “Reading the Eyes in the Mind” test [45]). Since people with and without ASD find it more difficult to read the facial emotional expressions of people with ASD [41], it is conceivable that further obscuring the amount of visible facial information could make the interaction even more arduous. This point may be particularly relevant to interactions between people with ASD and their unaffected family members. ASD is a highly hereditable condition with a complex genetic basis [46], and many unaffected relatives of children with ASD have been found to have subclinical autistic traits [47]. The parents of children with ASD may demonstrate subtle deficits in social communication and face processing [48,49].

Given these reports and considerations, the physical presence of smartglasses may affect social communication, and it may be sensible to attempt to minimize such facial obscuration to enhance social communication between people with ASD and their family members.

The presence of face-worn smartglasses may also influence social relationships, of the adults or children who wear them, as they alter a user’s facial appearance. Unlike many other assistive technologies, they are not easy to hide. Wearing smartglasses may not only alter how the user perceives the world but may alter how the world perceives the user. Facial appearance plays a key role in determining how people interact with one another [50], including whom they help, hire, or want to date [51]. Human faces may also be judged based on their symmetry, a marker of attractiveness and an indicator of optimal developmental outcome despite environmental stressors [52]. Greater facial symmetry has been linked to increased perceived trustworthiness and a decreased risk of being bullied [53]. Facial symmetry may be perceived as demonstrating genetic quality and therefore suitability of an individual as a mate [52], while facial asymmetry may be a predictor of long-term psychological, emotional, and physiological distress [54]. Users of smartglasses that are asymmetrical, such as those that are monocular, could be perceived as being less attractive and trustworthy due to the aforementioned principle of evolutionary psychology. By extension, “asymmetric” smartglasses users may also be at greater risk of bullying [53]. On the other hand, smartglasses that are asymmetrical may obscure less of the wearer’s face from the view of others. As discussed earlier, maximizing how much of the face is visible may help facilitate social communication. Even nontechnological face-worn glasses are associated with impaired interpersonal relationships: for example, wearing prescription glasses or having a history of using eye patches has been associated with a 35% increase in the likelihood of receiving physical or verbal bullying [55].

### Smartglasses in the Context of Child Development

The perceptual impact of smartglasses and their ability to augment a child’s cognitive and emotional functioning may have a central and influential role in childhood development if we consider Bronfenbrenner and Ceci’s bioecological model [56] and Bronfenbrenner’s earlier ecological systems theory [57]. According to the bioecological model, children are active participants in their environments and they have unique bidirectional interactions with each of their contextually separate environments, including home and school. This model places increased emphasis on the cognitive, emotional, and physical attributes of the child in his or her development and in how the child and environments interact with one another. As outlined in Bronfenbrenner’s ecological systems theory [57], the school environment, like the home environment, is one of the most intimate and influential environments affecting childhood development, as it lies in the child’s microsystem. When we consider that smartglasses may enhance the cognitive and emotional functioning of children within their microsystem, we can see that they may have a highly influential role in child development. Even within the microsystem, the contextual differences between the most intimate of environments may affect a child’s view toward using assistive technology. Research has shown that children have different attitudes and levels of enthusiasm toward using assistive technology depending on whether they are asked to use it at home or at school [32].

Furthermore, use of smartglasses by future school-age children and adolescents should prompt a discussion of Erikson’s 4th and 5th psychosocial stages [58]. Erikson identified a range of psychosocial developmental stages from birth through death. School-age children experience Erikson’s 4th psychosocial stage, described as a psychosocial crisis of industry versus inferiority. A child in this stage is often expected to learn and demonstrate new skills, productively complete tasks, and meet the expectations of parents and teachers. During this stage, a child becomes aware of his or her abilities and the abilities of his or her peers. A child who cannot master these expected skills risks a sense of inferiority and failure. The potential impact of smartglasses on this developmental stage is not known. They may aid children in successfully mastering this psychosocial stage by allowing them to be productive and giving them a sense of achievement. There is also a risk that children may feel inferior if they feel that without the smartglasses they are incompetent or if they feel ridiculed for wearing such devices. Each child may face a unique situation based on his or her own personal attributes and the support received from key people such as teachers, parents, and peers. This highlights the importance of ensuring that these key people are familiar with smartglasses technology and understand its capabilities and functionality.

Following this stage is Erikson’s 5th psychosocial stage that occurs in adolescence, described as a psychosocial crisis between identity versus role confusion [58]. Adolescence is a time of tremendous biological and psychological change [59], and during this stage individuals seek to define their role in the world, seeking to address the existential question, who am I and what can I be? Individuals will try to find like-minded social groups, focus on relationships with peers, and pursue a sense of belonging. Many questions remain unanswered about how smartglasses may impact people with ASD during this stage, especially given the many social challenges people with ASD encounter during this transition from childhood to adulthood [60]. How will ASD and these technologies define the individual? Will these technologies help individuals to find their purpose or hinder them? The impact of such technology may depend on smartglasses’ physical attributes, their impact on social relationships, or individual person characteristics (as discussed above within the scope of the bioecological model [56]).

Learning happens continuously in childhood, and the use of smartglasses technology may provide a digital means of enabling learning to occur, as in Vygotsky’s zone of proximal development (ZPD) [61]. Vygotsky originally described his ZPD as being “the distance between the actual development level as determined by independent problem solving and the level of potential development as determined through problem solving under adult guidance or in collaboration with more capable peers” [62]. These smartglasses designed as assistive technologies may allow children to undertake and learn tasks that they would have found impossible or very difficult to do independently. A child with ASD normally has a number of challenges in being in the ZPD, such as becoming overwhelmed with new experiences, struggling with transitions in environment or activities, and coping with sensory stimuli [18]. Sensor-rich smartglasses may be of particular utility here in that they can be used, with the right software, to monitor the behavioral and physiologic functioning of a child. For instance, they can be transformed by software to be able to detect when children are under- or overstimulated and to accordingly adapt the learning experience in real time to keep a child engaged and in the ZPD [8].

### Victimization, Socialization, and the School Environment

School-age children with ASD are at risk of being stigmatized [63] and being victims of bullying [64] for multiple reasons. They have different developmental trajectories that may put them at greater risk of victimization than their neurotypically developing peers, especially when they have challenges in social skills and communication [64]. They may struggle to recognize social cues and develop relationships with their peers, impeding their ability to be better integrated by the community [65-67]. Bullying may be particularly problematic at school, where academic and social factors may be a source of considerable stress, anxiety, and mental health concerns in children [68-70]. A school represents not only an academic establishment but a complicated and highly social environment. Children in schools often balance interpersonal relationships with peers and staff, complex social hierarchies, and school rules that can dictate the most basic elements of children’s day (whom to play with, where to sit, and when to talk to others [65-67,71]). Some reports have suggested that children with ASD have inherently low motivation or desire to join social groups, but recent evidence indicates this is not the case and many have a strong desire for acceptance [72-74]. Therefore, it is important to consider the acceptability and design of any assistive device in the population, given the risk of stigma and social isolation [30]. This is especially true for a device that is worn on the face.

## Methods

### Study Outline

We gave 8 children with ASD an opportunity to try the Glass smartglasses in a controlled, recorded environment and to explore its features, usability, and visual characteristics. We observed and recorded the interaction of the children with the device. We also conducted a postsession semistructured interview with the children and their caregivers, who accompanied the child and observed the whole session. Our sample represented a broad age range and severity spectrum of ASD.

### Institutional Review Board Statement

The use of the Brain Power Autism System running on multiple head-worn computing devices by children and adults with autism was approved by Asentral Inc Institutional Review Board, an affiliate of the Commonwealth of Massachusetts Department of Public Health. The study was performed in accordance with relevant guidelines and regulations.

### Participants

Eight children with clinically diagnosed ASD and their caregivers were entered into this study. The participants represented a wide range of school-aged children, ages 6.7 to 17.2 years (mean 11.7 [SD 3.3] years), including 7 males and 1 female. Participants were recruited from a user research database created from Web-based research interest forms. Written consent for study participation was obtained from the legal guardians, and children from age 7 to 17 years provided written assent. In this report, every participant was accompanied by a parent or guardian caregiver to the session, and participants and caregivers could exit the session at any time and for any reason. It was explained that the main aim of the study was to understand the acceptability and usability of modern smartglasses technology in children with ASD.

Caregivers rated the participant level of overall ASD functioning according to a subjective 7-point scale (1=lowest-functioning/severe to 7=highest-functioning/mild). Caregivers also rated speaking ability on a similar scale (1=nonspeaking to 7=fully conversational). Participants represented a large range of overall ASD functioning (range 4 to 7 out of 7; mean 5.6 [SD 1.1]) and speaking ability (range 4 to 7 out of 7; mean 5.5 [SD 1.3]).

### Data Collection Procedure

Participants and their caregivers were orientated to the testing room where they had an opportunity to learn about the Glass smartglasses and to physically wear and use them. They were provided with any assistance they required to properly place the smartglasses on their heads and align it with their eyes, although little assistance was needed. They were able to use any of the apps on the smartglasses. Testing sessions were recorded via video and photographs. All participants and/or caregivers gave written consent for their images and video to be used in current and future research analyses.

Following the testing, participants and caregivers went into a separate room where they were questioned about their experience as part of a semistructured interview. The participants were asked to compare their experience of Glass with previously tested assistive devices and gamified apps related to ASD. As previously noted, the participants were recruited from a research database for technology-related studies in ASD, and all had seen and tried the original Google Glass. Participants were asked if they became stressed when using the device and if the session was an overwhelming sensory or emotional experience for them. The questions were adapted or simplified based on the child’s speaking ability and were repeated if needed. Study staff interacted with the child and caregiver and spent time ensuring the questions were understood, considered, and accurately answered.

Participants were then asked whether they would consider wearing and using the device for 1 hour each day in their school and separately asked the same question about using the device at home. The caregiver was also interviewed in order to rate whether they felt the experience was fun for the participant and whether they felt the experience with the smartglasses went better than they had expected.

### Exclusions

Individuals who had a known history of epilepsy or seizure disorder were not asked to take part in this study. Individuals who had any uncontrolled or severe medical or mental health condition that would make participation in the study predictably hazardous were also not invited to participate.

## Results

**Figure 2:**
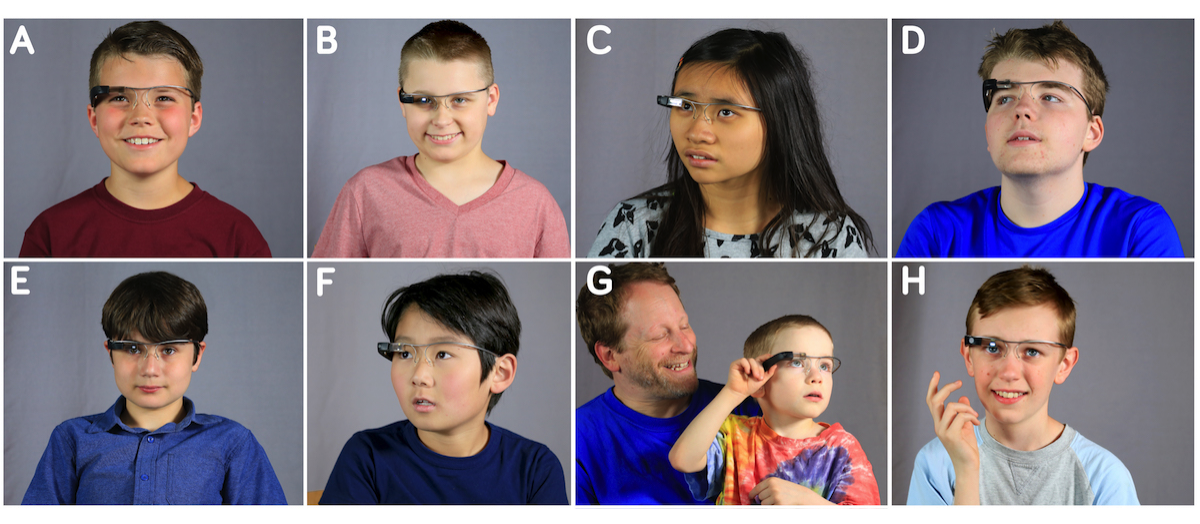
Children on the autism spectrum using and exploring the Glass Enterprise Edition device during a testing session at Brain Power. Each of the 8 participants, who represent the entire range of school ages, range from mild to moderate autism severity, and demonstrate a wide breadth of speaking ability (from moderate impairments in speech to being fully conversational), rated Glass Enterprise Edition as desirable to wear on their heads and use daily in the often-complex social environment of school and at home.

All 8 children, who represented the full range of school ages (6 to 17 years), successfully wore, interacted with, and explored one or more Glass smartglasses (Figure 2). The smartglasses were loaded with a suite of assisted-reality apps for social-emotional learning and self-coaching related to brain-based challenges and needs, as discussed elsewhere [8]. Participants explored the devices at their leisure, putting them on and taking them off and exploring the style, size, weight, shape, and features such as foldability, and spoke out loud in some cases (children with greater speaking ability) about their observations and questions. All children successfully transitioned to the interview room, where they responded to questions by the experimenter, accompanied and assisted by their caregivers as needed. There were no negative effects reported or observed.

All participants noted that they did not feel stressed (8/8, 100%, Table 1) or have an overwhelming sensory or emotional experience when using the smartglasses (8/8, 100%). The participants all reported that they would be agreeable to using the smartglasses in both home (8/8, 100%) and school settings (8/8, 100%). Caregivers reported no concerns with the children using the smartglasses, and all caregivers reported that their child appeared to have fun using the device (8/8, 100%). The majority of caregivers felt the interaction of the child with the smartglasses went better than they had expected (6/8, 75%; Table 2). Of the remaining 2, 1 parent said that the experience had proceeded “as expected” and another answered the question conversationally but without a direct response, so the response was not tabulated as a yes but as an undetermined.

**Table 1:** Participant responses following use of smartglasses.

Question	Yes n (%)	No n (%)	Neutral or undetermined response n (%)
Would you wear the smartglasses for 1 hour each day at school?	8 (100)	0 (0)	0 (0)
Would you wear the smartglasses for 1 hour a day at home?	8 (100)	0 (0)	0 (0)
Did you feel stressed while wearing the smartglasses?	0 (0)	8 (100)	0 (0)
Did you feel overwhelmed (emotionally/sensory)?	0 (0)	8 (100)	0 (0)

**Table 2:** Caregiver responses following use of smartglasses.

Question	Yes n (%)	No n (%)	Neutral or undetermined response n (%)
Was it fun for your child to use Glass?	8 (100)	0 (0)	0 (0)
Did the experience go better than you anticipated?	6 (75)	0 (0)	2 (25)

## Discussion

### Principal Findings

Smartglasses are an emerging technology that could hold much promise as an assistive technology for children and young adults with ASD. It is important to seek the opinions of children with ASD and their caregivers when considering the use of a new assistive device. This is especially true of smartglasses given their high level of visibility, prior reports of negative social perceptions, and the potential interplay of such devices with social communication and child development. Children with ASD and their caregivers may be particularly discerning about factors that could impact the use and social acceptance of such technologies in educational settings such as schools and in the home environment.

The results demonstrate that Glass was acceptable and desirable by all participants, who spanned the full range of school ages (6 to 17 years). It was encouraging to find that all 8 school-aged children with ASD felt that using these smartglasses was not a stressful experience and denied being overwhelmed in a sensory or emotional way. Additionally, it was also promising to see that all of the children expressed a willingness to use these devices in both school and home settings. Caregivers noted that children had fun using the device, and most caregivers felt their expectations of how the children would interact with the smartglasses were surpassed.

These results are important for a number of reasons. Children with ASD are frequently not involved in providing design or usability feedback to interventions and technologies developed for them. Involving children when choosing an assistive device is crucial to ensure that the device is socially appropriate for the environment, which will likely lead to greater compliance in wearing the device. It also appears that these children are accepting of new technologies, even on relatively uncommon and highly visible platforms such as head-mounted computers. The children who participated in this study were more open to using Glass in a public environment than many adults have been [22]. With this in mind, it will be equally as important to ensure caregivers and peers in the child’s microsystem are accepting of the assistive technology [57], as their opinions will likely sway a child’s enthusiasm toward the device. Many children in this study mentioned favoring Glass because of its unobtrusive, sleek design; having a device that is less noticeable and designed to be “cool” may help with its social acceptance and may not carry the stigma of assistive technology with it. The desirability of Glass in this case was predicated on a prediction of social acceptability (colloquially, the “cool factor”) in a social situation. Many factors may be included in a participant’s prediction of the cool factor of a device. Such factors may include unobtrusiveness, lightness, futuristic look, comfort, ease of storing, ease of transport, durability, ruggedness, styling, ability to give others experiences they could not otherwise have (conferring to the child an ability to control a social situation in a positive way), ability to initiate a conversation with decreased anxiety over selecting the topic of the conversation (ice-breaker), and more.

### Limitations

The unanimous willingness of participants to wear the smartglasses in school is also important. The school setting is a place of high risk relative to social integration and stigma that could result from an undesirable or socially inappropriate device or behavior. This is one reason we chose the question of acceptability of the device at school as a high-bar test for how desirable and acceptable this new device may be. However, a limitation of this work is that we asked for the opinion of the target users, and such an opinion is necessarily based on a prediction. It may be hard to predict how a device or behavior will actually be received in the complex and changing social hierarchy of a school environment. Additionally, children with ASD may have extra challenges in predicting the emotional reactions and behaviors of their classmates, especially if they are in an integrated school environment with neurotypical or typically developing children their same chronological age. For all these reasons, further research is needed to test the acceptability within school environments.

### Conclusions

These results suggest that a smartglasses platform may be an acceptable base for assistive software apps that could promote self-sufficiency. For instance, they may have a desirable new platform for gamified, social-emotional self-coaching apps based in neuroscience and artificial intelligence that have been deployed on other head-worn computer platforms [8]. The results are promising at a broader level for those who wish to use or develop apps that harness the unique features of this family of devices, such as their ability to allow the user to be heads-up, hands-free, and able to perceive and engage with the world around while receiving additional assistance. The results suggest that the newest entrant into the still-emerging family of devices may be well received, at least by some discerning populations. Further research is clearly needed to address these and more limitations or open questions of this work. This report represents part of a larger, ongoing research initiative.

This paper represents the first published work, to our knowledge, using Glass (Enterprise Edition). It also represents the first published use of Glass as an assistive or assessment device for people with different abilities or intellectual disabilities or challenges. This work extends our previous research on the use of the original Google Glass as an aid to people with ASD [8].

## Abbreviations

ASD: autism spectrum disorder
Glass: Glass Enterprise Edition
ZPD: zone of proximal development
